# The Benefits of Cognitive Therapeutic Exercise in Symptomatic Arnold–Chiari Syndrome Type I: A Case Report on Gait, Balance, and Pain Management

**DOI:** 10.3390/jcm13185502

**Published:** 2024-09-18

**Authors:** Adriana Tisano, Angelo Alito, Rita Ragonese de Gregorio, Adele Campo, Giuseppe Santoro, Demetrio Milardi, Filippo Cavallaro, Francesca Cucinotta

**Affiliations:** 1 Department of Clinical and Experimental Medicine, University of Messina, 98125 Messina, Italy; atisano@unime.it (A.T.); ritaragonesedg@gmail.com (R.R.d.G.); adycampo96@gmail.com (A.C.); 2 Department of Biomedical, Dental Sciences and Morphological and Functional Images, University of Messina, 98125 Messina, Italy; giuseppe.santoro@unime.it (G.S.); dmilardi@unime.it (D.M.); 3 Physical Rehabilitation Medicine Department, University Hospital A.O.U. “G. Martino”, 98124 Messina, Italy; filippo.cavallaro@unime.it; 4 IRCCS Centro Neurolesi Bonino-Pulejo, 98124 Messina, Italy; francesca.cucinotta@irccsme.it

**Keywords:** Arnold–Chiari, Chiari malformation, Chiari malformation type 1, clinical outcome, cognitive therapeutic exercises, neurological rehabilitation, rehabilitation

## Abstract

Background: Chiari malformation is a rare congenital condition in which the cerebellar tonsils herniate through the foramen magnum, causing symptoms related to compression of the surrounding structures. Rehabilitation plays a key role in the pre- and post-operative management of the syndrome, as it can improve strength, range of motion, motor coordination, pain management, and performance of activities of daily living. Methods: This article presents the case of a 43-year-old woman with Chiari malformation 1B who underwent resection of the filum terminale. She presented as an outpatient at the University Hospital “G. Martino” in Messina, complaining of difficulty walking, balance problems, lumbar pain, and heaviness in the lower limbs. Following a multidisciplinary assessment, she underwent an 11-month rehabilitation protocol based on cognitive therapeutic exercise. Results: The patient achieved significant improvements in pain, mental confusion, and quality of life after treatment and at the 12-month follow-up. Conclusions: The results of this study highlight the significant benefits of cognitive therapeutic exercises in Chiari malformation, with improvements in several key areas, including quality of life, pain management, and ability to perform activities of daily living.

## 1. Introduction

Chiari malformation (CM) is a rare congenital condition (ORPHA code: 268882) consisting in the herniation of the cerebellum through the foramen magnum, resulting in an abnormal pressure on the brain and spinal cord [1]. CM epidemiology is unknown and not fully investigated, with a global prevalence ranging from 1.9 to 8.4/100,000 [2,3].

CM is classified into four main types (CM I–IV) based on the severity and the anatomical features involved [1]. Type I evolves from an anomalous closure of the notochord, and it is divided into CM1A and CM1B, depending on the presence of Syringomyelia. Type II is characterized by the tonsillar herniation associated with spina bifida. Types III and IV come from extremely severe and ultra-rare embryonic anomalies and are unrelated to types I and II [4]. A form of “acquired” CM is also known, consisting in the herniation of the cerebellar tonsils secondary to space-occupying lesions, such as hydrocephalus, brain tumors (e.g., meningioma 36%), and arachnoid cysts (32%), or after traumatic brain injuries to the posterior cranial fossa [5].

The gold standard for diagnosis is magnetic resonance imagining (MRI), with a minimum descent of the tonsils of 5 mm to define the pathology; a descent less than 3 mm is considered a physiological variant; a finding between 3 and 5 mm defines an uncertain, borderline case that needs further investigation in symptomatic patients or when combined with syringomyelia (or other anomalous findings) [6].

CM syndrome can present with a variety of non-specific symptoms, due to brainstem and cerebellar involvement, and is defined by the presence of at least two symptoms, such as headache, especially at occipital/suboccipital level and worsened by coughing, sneezing, sudden postural changes, and physical activity [7]; nystagmus [8]; dysphagia [9]; central and obstructive sleep apnea [10]; and ataxia [2]. Motor and sensory changes can also occur, leading to weakness, spasticity, and impaired fine motor coordination, as well as numbness, tingling, or loss of sensation [11,12]. Other minor manifestations may be present, such as balance impairment, hypo- or hyperacusis, or dizziness [13].

Recent data from the literature highlighted reduced performance in attention, memory, visuospatial ability, and processing speed in both pediatric and adult CM patients [14].

Pharmacological treatment aims to modulate pain and spasticity, and therapeutic exercises, stretching, and modalities (e.g., TENS) can play a role in pain management and mobility impairment [15,16].

In addition, neurorehabilitation techniques such as proprioceptive neuromuscular facilitation, Bobath’s method, and cognitive therapeutic exercise (CTE) may be used for this condition [17,18,19].

CTE is a rehabilitation technique introduced by Carlo Perfetti, originating from Anokhin’s theory, based on sensory retraining and focusing on the joint perceptual space during task performance [17,20,21]. According to Perfetti, movement is a complex interaction between the subject and the environment, involving cognitive functions such as perception, memory, attention, language, and decision-making [22]. This approach relies on neuroplasticity, and by engaging patients in exercises that require both cognitive engagement and physical movement, CTE aims to stimulate these neural pathways to improve proprioception and exteroceptive perception with visual, auditory, and tactile stimuli in the peri-personal space [23].

According to the international consensus document resulting from the Interregional Syringomyelia and Chiari Consortium (CSC) in 2019, the treatment of symptomatic CM is based on surgical approaches [1]. The foramen magnum decompression (FMD) is appropriate in symptomatic forms of CM1-A without hydrocephalus and, indeed, not necessary in asymptomatic isolated CM1. FMD is the surgical gold standard and can be performed as a bone-only decompression consisting of opening the foramen magnum with additional duraplasty or even tonsillectomy to allow cerebrospinal fluid to flow and reduce pressure on the nerve structures [1,24].

Another surgical technique described in the management of CM1 patients, although not included in international guidelines, is excision of the filum terminale (FT), consisting in the resection of FT after neurophysiological identification [25]. Even if some authors have suggested a potential role in CM1 symptom management, there is no strong evidence regarding the real efficacy [25,26].

The aim of this case report is to describe a single patient with CM1, who benefited from CTE following surgical FT resection. This case report followed the CARE guidelines for case reports [27].

## 2. Case Presentation

**Figure 1:**
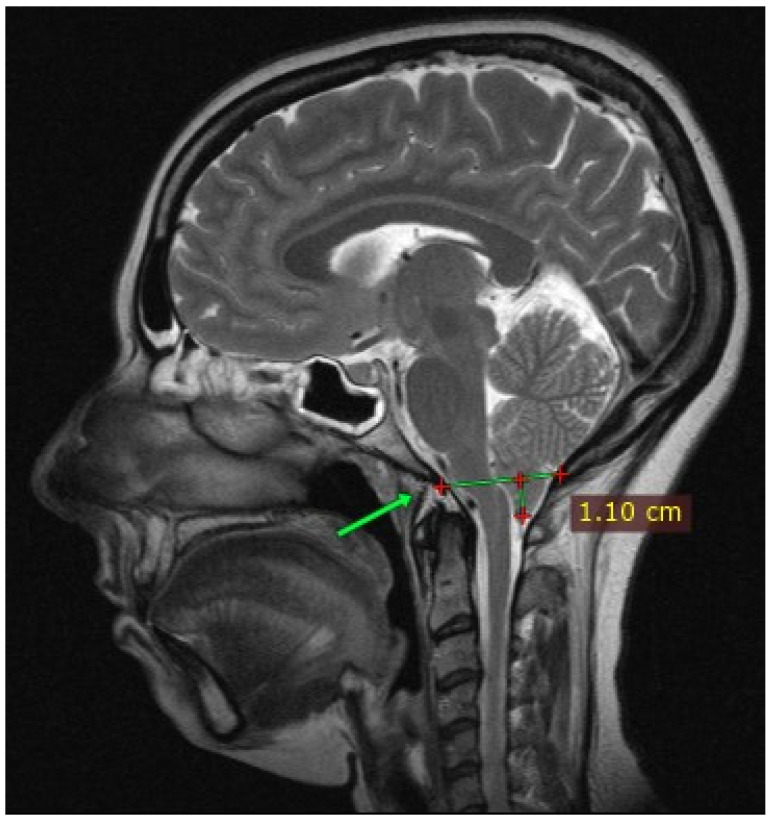
MRI scan showing a Chiari malformation characterized by the inferior displacement of the cerebellar tonsils through the foramen magnum. The green line, marked by the green arrow, is the McRae line.

The authors present the case of a 43-year-old woman with CM1 syndrome (ICD-10 code Q07.0). The patient reported occasional headaches since the age of 16, of varying intensity, with nausea, blurred vision, photophobia, retro-ocular pain, sonophobia, hyperacusis, vertigo, dysphagia, difficulties with speech and concentration, anxiety, emotional lability, and paresthesias in the right hemifacial region. Cervicalgia and low back pain, with paresthesias in the limbs, feelings of weakness, with difficulty walking and climbing stairs, were also reported. In May 2012, the family doctor recommended a brain and cervical MRI because of recurrent cervical pain symptoms, and CM1A was diagnosed, which showed a 14 mm dislocation of the cerebral tonsils and a reduction in the size of the posterior fossa without disks alterations (Figure 1). In 2014, the patient underwent a consultation at a specialized CM center, which did not indicate surgical intervention.

**Figure 2:**
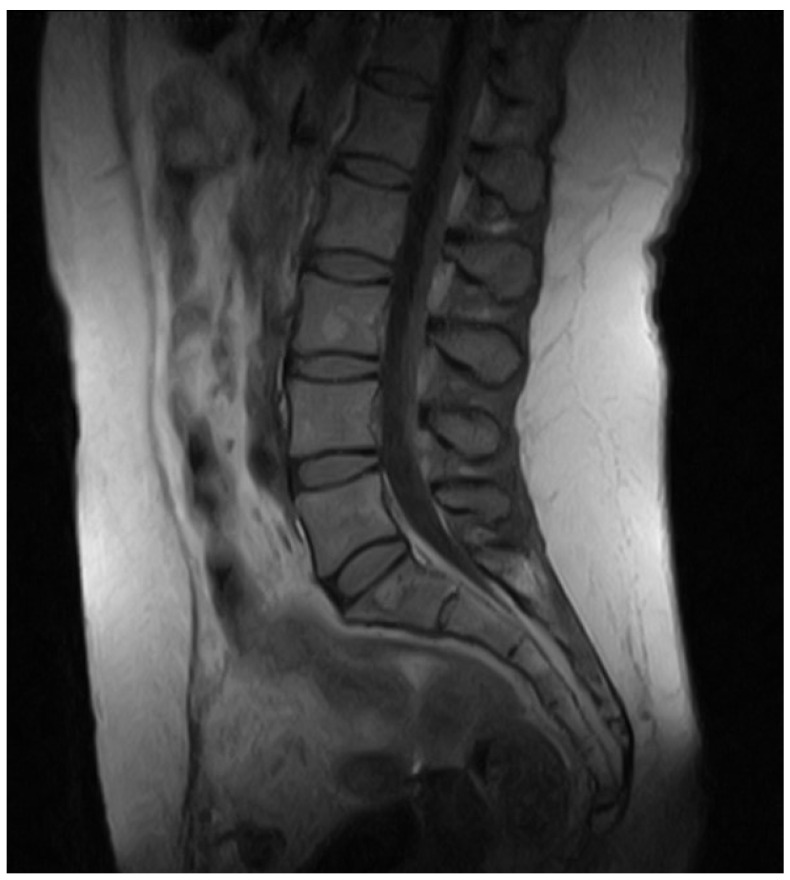
MRI scan showing angiomas on the vertebral bodies of L1, L3, and L5, with normal disc thickness performed in June 2020.

During the first months of 2020, the patient complained of rapid and progressive clinical deterioration, with widespread pain, dysphagia, balance, and motor coordination problems. To rule out lumbar discopathy or instability, the patient underwent MRI of the lumbar spine, which showed the presence of small angiomas on the vertebral bodies of L1, L3, and L5, with normal disc thickness (Figure 2).

**Figure 3:**
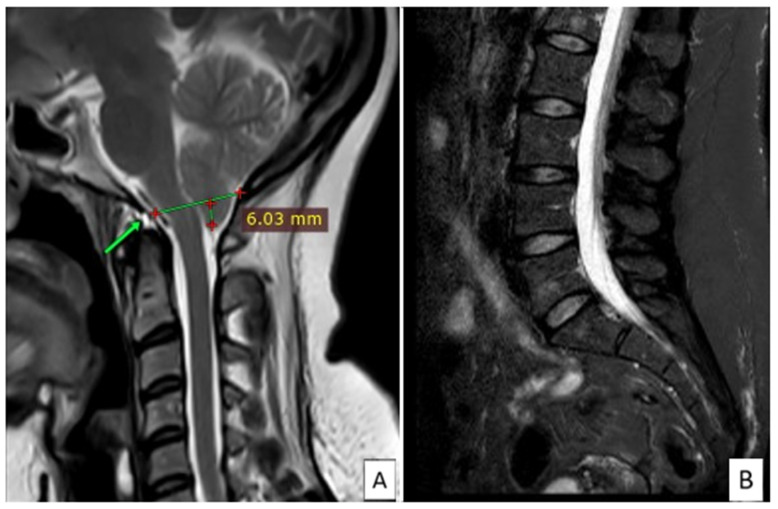
Post-surgery MRI evaluation of the brain with the McRae line marked by the green arrow (**A**) and the lumbar spine (**B**).

In June 2020, she had to use crutches because of weakness and tremor of the lower limbs, involuntary movements of the lower limbs during sleep, leg fasciculations, and instability when walking. Psychologically burdened by the clinical condition and after personal online research, she decided to undergo a specialist examination at a center specialized in CM and syringomyelia. After a clinical evaluation, the physicians diagnosed “neuro-cranio-vertebral syndrome, filum disease, and CM1” and recommended surgery after a preoperative study to exclude contraindications. The FT resection surgery was performed in August 2020, with partial recovery of symptoms but residual heaviness and paresthesia in her legs, referred to as “wearing ice knee pads”. The follow-up lumbar MRI showed the previously known small angiomas on L1, L3, and L5, normal disc thickness, and a brain MRI with a herniated tonsil of about 6 mm (Figure 3).

**Figure 4:**
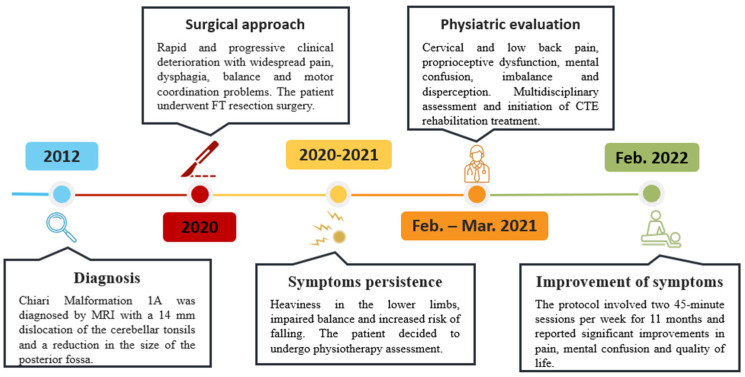
Clinical history and rehabilitation program timeline detailing the progression from initial diagnosis and medical interventions to personalized rehabilitation plan. Template by PresentationGO—www.presentationgo.com (accessed on 21 July 2024).

The clinical condition dramatically worsened the patient’s risk of falling, and in February 2021, the patient presented as an outpatient at the Physical Medicine and Rehabilitation Unit of the University Hospital “G. Martino” of Messina. A timeline representation of the clinical history is provided in Figure 4.

The initial assessment was carried out by a physiatrist and the patient presented with dorsal hyperkyphosis, low back pain, limited range of motion and painful movements of the cervical spine, contracture of the neck muscles, and proprioceptive changes, especially in the lower limbs. There were no reports of changes in muscle tone or strength. She also reported a subjective feeling of mental confusion and imbalance, with difficulty avoiding obstacles when walking and dysperceptions.

**Figure 5:**
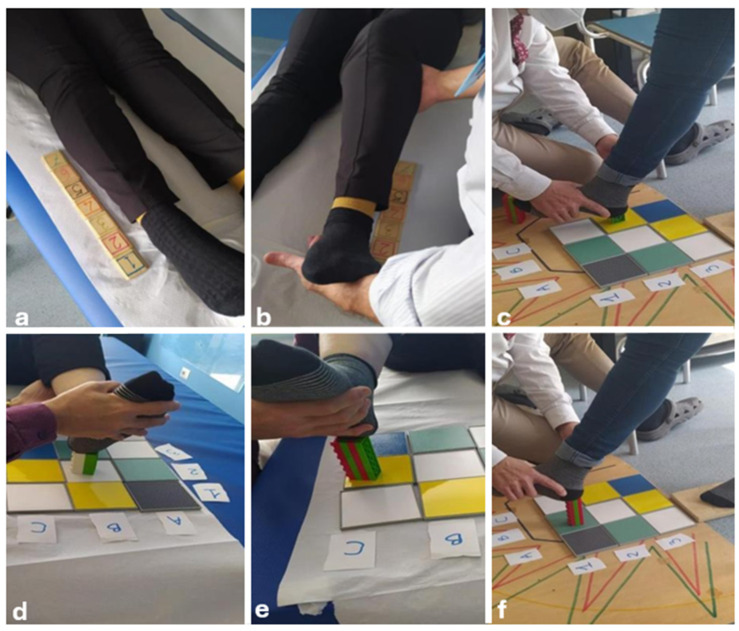
First phase: physiotherapist-guided exercise consisting of identifying different positions by sliding the heel (**a**,**b**) or the height and shape of the brick used (**d**,**e**), in order to stimulate sensory and executive memory, space management memory, the connections between the temporal and frontal areas of the CNS, and the selective cerebellar areas responsible for controlling coordination. In addition, in a standing position with eyes closed, the patient had to identify different leg positions, combined with proprioceptive stimulation (**c**,**f**). Each set of each exercise consisted of ten repetitions, performed with the right and left lower limbs, respectively. During the rest period, the patient was asked about difficulties during the exercises and about body sensations.

**Figure 6:**
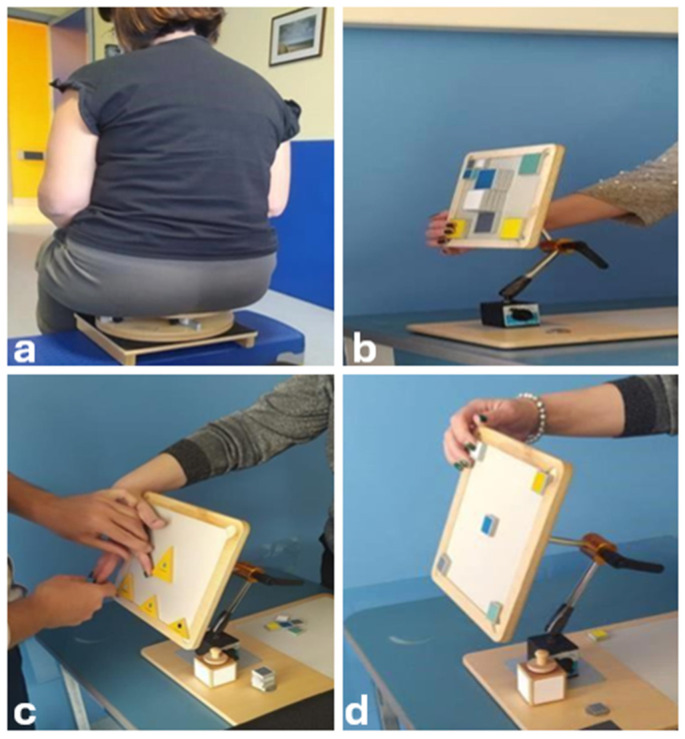
Second phase exercises aimed to stimulate a cognitive recognition process. A “golden pillow”, a highly unstable adjustable support, was used in a seated position, with the aim of detecting the position of the springs through a tilting movement, integrating afferent information from different areas of the CNS (**a**). A magnetic board was used to apply magnetic elements with different surfaces, materials, shapes, and sizes. The patient’s task was to identify the magnets of different colors and surfaces and their position on the board, first in a sitting and then in a standing position (**b**–**d**). These exercises aimed to improve the acquisition of motor imagery, the management of space, and the refinement of the tactile function using pathways between the temporal, occipital, and frontal areas of the CNS.

**Figure 7:**
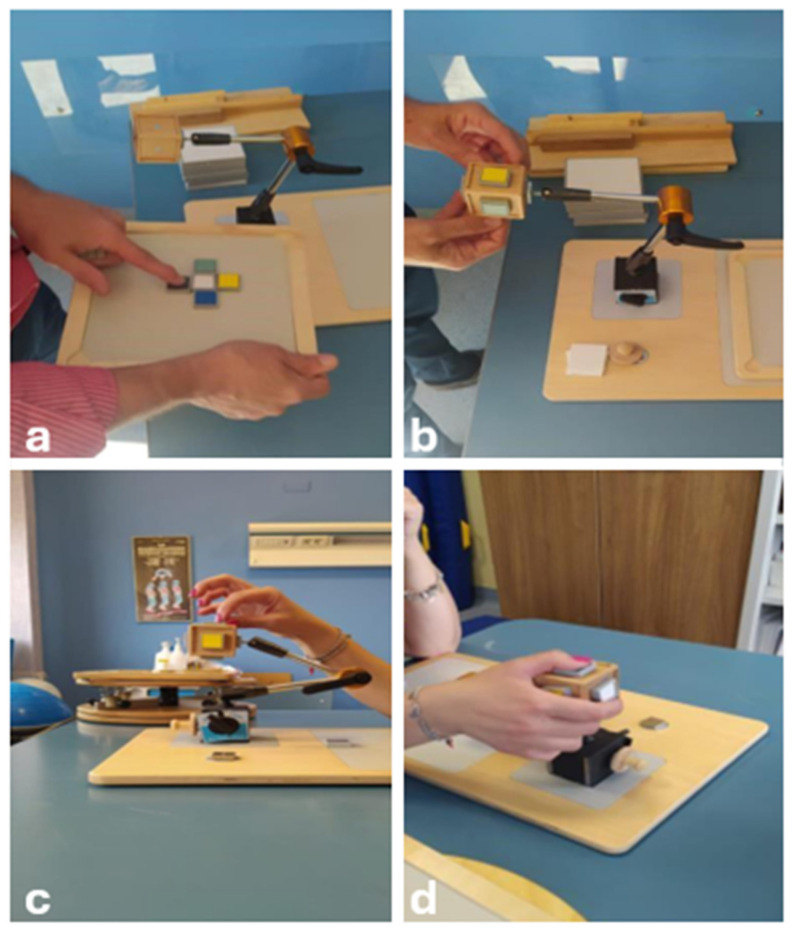
Third phase exercises: the patient learned to adapt her movement to the proposed perceptual hypothesis and to overcome elementary motor patterns. The patient’s attention was exclusively focused on evaluating the discrepancy between the performed scheme and the one proposed by the physiotherapist. The aim was to identify (**a**) and rearrange the magnets arranged on the five faces of a cube and to reproduce the correct order of the different faces on the magnetic table with the eyes closed and both hands (**b**–**d**). Each series consisted of five different combinations.

Following a multidisciplinary assessment, she started a rehabilitation protocol based on the CTE approach, with the aim of reducing pain symptoms, learning correct ergonomics in daily activities, and improving motor coordination and balance. The program included a phased approach, gradually increasing the difficulty of exercises (Figure 5, Figure 6 and Figure 7). The first physiotherapy session focused on educating the patient about the disease and the deficit she had, as well as her perception of her body in space, and was fundamental in establishing a trusting relationship between the therapist and patient. The exercise protocol started with simple passive exercises, to reach a higher level of motor and cognitive skills in a three-level difficultly protocol. After two months, the patient complained of significant upper limb pain and altered sensation, particularly in the right upper limb, with a tingling sensation from the right shoulder to the index finger of the right hand, so the protocol was redesigned to include upper limb exercises.

Functional assessment was performed at the first physiatric evaluation (T0), at the end of treatment (T1), and at 12 months (T2). The Short Form Health Survey (SF-36) was used to measure quality of life [28], the NRS was used to measure pain [29], the Chicago Chiari Outcome Scale (CCOS) was used to assess clinical outcome [30], the Italian version of the Chiari Symptom Profile (CSP) was used to measure CM symptoms and their impact [31], and the impairment due to dysperceptions of the lower limbs was measured using a visual analogue rating scale ranging from 0 (no disability) to 10 (maximum disability).

The patient began the rehabilitation program in March 2021 and completed two 45 min sessions per week for 11 months, reporting a significant improvement in pain, mental confusion, and quality of life. She also reported an improved confidence in movement involving the spine and the limbs, and her gait was faster, with no difficulties in avoiding obstacles. Her balance was better than at the start of the rehabilitation program, with a reported reduced risk of falling.

The first phase of the protocol was based on a passive exercise in which the patient had to recognize a joint position chosen by the therapist, to stimulate the sensory, spatial, and executive memory, moving from voluntary (cortical) control to an automatic (subcortical) stimulation of the connections between the temporal and frontal areas of the CNS and the selective cerebellar areas.

After this phase, the patient reported improved body space management and proprioception, with a subjective sense of balance enhancement, and began to experience a reduction in pain.

The second phase required a higher level of patient participation and was aimed at the acquisition of motor image memory and the management of space, as well as the refinement of tactile function involving pathways and connections between the temporal, occipital, and frontal areas of the CNS.

During this phase, the patient felt an increase in control of complex movements and execution of motor schemes, notable pain reduction, and limb movements were faster, which also had a positive effect on mood.

In the third level exercises, the patient learned to adapt the movement to the proposed task without focusing on the previous components. The patient’s attention was exclusively directed to evaluating the discrepancy between the performed scheme and the proposed one. The aim was to overcome elementary motor patterns.

At the end of the treatment, the patient was able to apply the improvements gained from the treatment to daily activities, with improved quality of life, balance, and gait.

A comprehensive view of the results is provided in Table 1.

## 3. Discussion

The traditional therapeutic approach to CM1 can be both surgical and rehabilitative in symptomatic patients. In this case report, in March 2021, one year after the FT resection, the patient underwent an 11-month rehabilitation program according to the CTE method, which showed an almost complete resolution of the complained symptoms, such as balance and walking disorders, paresthesia, low back pain, and headaches, leading to a significant improvement in daily activities, quality of life, and mental health status.

CTE targets neurological disease by engaging patients in tasks that combine mental processing with physical activity. It is based on the principle that regular, targeted exercise can help mitigate decline in cognitive and motor skills by improving brain connectivity and promoting compensatory mechanisms [17]. This therapeutic approach emphasizes the importance of a comprehensive understanding of brain function, recognizing that improvements in cognitive health can significantly influence motor recovery and vice versa [32].

The levels of CTE in neurorehabilitation can be understood as stages of increasing complexity and intensity, adapted to the patient’s progress in recovery. First-level exercises often do not require any active movement and are designed to improve control of movement and perception of the body in space without the aid of vision, focusing solely on joint and muscle proprioception. The patient must describe the amplitude and intensity of the movement to transform it into kinesthetic information, and the body can be considered as a receptor matrix capable of transmitting the necessary information to the central nervous system [33]. At this stage, muscular relaxation can lead to an improvement in pain through a positive feedback mechanism that inhibits the nociceptive afferences arising from muscular imbalances. In second-degree exercises, the patient can improve and integrate several cognitive and motor skills through kinesthetic awareness of the joint areas. Voluntary and dual-tasking movements require the patient to pay attention to the motor quality of the proposed movement. The higher-level exercises aim to restore higher level cognitive and motor functions, to promote the integration of complex cognitive processes [33].

CTE may be useful, not only in the treatment of CM1, but also in neurological and/or degenerative conditions such as stroke, facial paralysis, and musculoskeletal disorders in orthopedic rehabilitation [32,34,35,36].

The main aim of CTE is to restore adequate information transmission by activating cognitive processes. Movement allows information to be sent from the periphery to the cortex via different sensory fibers and, through constant repetition over time, favors the construction of new neural networks [32].

CTE can help reduce pain through a multifaceted approach that addresses both the physical and psychological aspects of chronic pain. In CM, chronic pain can lead to dysregulation of pain pathways, and the CTE program, through physical and cognitive exercises, helps to reverse these maladaptive changes by promoting positive neuroplasticity and modulating pain signals [2,37].

In addition, the cognitive aspect can help patients change their thoughts and beliefs about pain; by challenging negative and catastrophic thought patterns, patients can change their emotional and cognitive responses to pain, which can lead to a reduction in perceived pain intensity [38,39].

The concept of neuroplasticity reveals how neurons, although incapable of replicating, can reorganize themselves through dendritic sprouting or the arborization of neighboring neurons, allowing the formation of new connections [40]. Neuroplasticity is the mechanism that underlies learning and motor control, allowing motor patterns to recover after a damaging event [41,42].

Physiotherapy is an important component of CM rehabilitation, as it can improve strength, range of motion, motor coordination, pain management, and performance of activities of daily living, as demonstrated in a case report by Ingale et al., in which a 19-year-old patient with CM1B who had undergone initial surgery (FMD) underwent a 12-week physiotherapy program with benefit [43].

A similar study by Tiwari et al. reported the case of a 39-year-old woman with syringomyelia associated with CM1 who was treated with a physiotherapeutic approach for pain relief using TENS and cryotherapy, therapeutic exercises to improve ROM, progressive resistance exercises for strength, followed by postural correction exercises, and exercises for coordination and gait [44].

This study has several limitations. Firstly, it reported a single clinical case, which is difficult to generalize from. Secondly, we must consider the spontaneous improvement in symptoms after surgery, except for balance deficits, where the role of physiotherapy was certainly essential. Future research with larger sample sizes and longer follow-up periods would be beneficial to confirm these findings and to explore the long-term effects of CTE.

## 4. Conclusions

The results of this study highlight the significant benefits of CTE in CM. Through a structured program of cognitive and physical exercise, the patient experienced significant improvements in several key areas, including quality of life, pain management, and ability to perform activities of daily living.

The reduction in pain levels not only contributed to a more comfortable daily life, but also facilitated greater participation in daily activities, which in turn increased the patient’s sense of independence and self-efficacy. In addition, the cognitive components of the therapy appeared to have a positive impact on her mental health, further improving quality of life.

## Figures and Tables

**Table 1:** Patient clinical data. The table shows pre- and post-rehabilitation assessments, including measures of symptom improvement, functional ability, and quality of life, highlighting sustained improvements at 12-month follow-up.

	T0	T1	T2
SF-36 (%)			
Physical functioning	30	75	90
Role limitations due to physical health	0	75	100
Role limitations due to emotional problems	66.7	100	100
Energy/fatigue	15	60	70
Emotional well-being	44	84	84
Social functioning	37.5	75	87.5
Physical pain	0	67.5	100
General health	30	50	60
Health change	0	75	100
NRS (0–10)	8	3	1
CCOS (4–16)	9	10	14
CSP (0–228)	138	58	19
VAS (0–10) (dysperception)	8	3	2

CCOS, Chicago Chiari Outcome Scale; CSP, Chiari Symptom Profile; NRS, numerical rating scale; SF-36, Short Form Health Survey; VAS, visual analogue scale.

## Data Availability

The data presented in this study are available within the article.
